# New localization and function of calpain-2 in nucleoli of colorectal cancer cells in ribosomal biogenesis: effect of KRAS status

**DOI:** 10.18632/oncotarget.23888

**Published:** 2018-01-03

**Authors:** Marcelino Telechea-Fernández, Lucia Rodríguez-Fernández, Concha García, Rosa Zaragozá, Juan Viña, Andrés Cervantes, Elena R. García-Trevijano

**Affiliations:** ^1^ CIBERONC, Department of Medical Oncology, INCLIVA Biomedical Research Institute/University of Valencia, Valencia, Spain; ^2^ Department of Biochemistry and Molecular Biology, INCLIVA Biomedical Research Institute/University of Valencia, Valencia, Spain; ^3^ Department of Anatomy and Human Embriology, INCLIVA Biomedical Research Institute/University of Valencia, Valencia, Spain

**Keywords:** nucleolar calpain-2, serum starvation, colorectal cancer, subcellular localization, pre-rRNA

## Abstract

Calpain-2 belongs to a family of pleiotropic Cys-proteases with modulatory rather than degradative functions. Calpain (CAPN) overexpression has been controversially correlated with poor prognosis in several cancer types, including colorectal carcinoma (CRC). However, the mechanisms of substrate-recognition, calpain-2 regulation/deregulation and specific functions in CRC remain elusive. Herein, calpain subcellular distribution was studied as a key event for substrate-recognition and consequently, for calpain-mediated function. We describe a new localization for calpain-2 in the nucleoli of CRC cells. Calpain-2 nucleolar distribution resulted dependent on its enzymatic activity and on the mutational status of KRAS. In KRASWT/- cells serum-starvation induced CAPN2 expression, nucleolar accumulation and increased binding to the rDNA-core promoter and intergenic spacer (IGS), concomitant with a reduction in pre-rRNA levels. Depletion of calpain-2 by specific siRNA prevented pre-rRNA down-regulation after serum removal. Conversely, ribosomal biogenesis proceeded in the absence of serum in unresponsive KRASG13D/- cells whose CAPN2 expression, nucleolar localization and rDNA-occupancy remained unchanged during the time-course of serum starvation. We propose here that nucleolar calpain-2 might be a KRAS-dependent sensor to repress ribosomal biogenesis in growth limiting conditions. Under constitutive activation of the pathway commonly found in CRC, calpain-2 is deregulated and tumor cells become insensitive to the extracellular microenvironment.

## INTRODUCTION

Calpains are Ca^2+^-dependent cysteine proteases belonging to a super family of 15 members identified to date. Among them, two isoforms, calpain-1 and calpain-2 (also known as μ-calpain and m-calpain, respectively) are distributed ubiquitously in mammalian tissues [1]. Both calpains form heterodimers composed of a unique catalytic subunit (80KDa) and a common regulatory subunit (30–29KDa) named calpain-4 [2].

Calpains are known as processing enzymes rather than proteases because in contrast to other proteases, the end-product of their enzymatic activity is not substrate degradation, but a processed-substrate which could then acquire a different function or ways of regulation [2, 3].

Although the specificity-determinants of substrate recognition remain elusive, more than 200 substrates are potentially recognized *in vitro* by calpains [3, 4]. The large number of calpain-substrates explains the variety of physiological processes they are involved in [3], going from the modulation of cell survival and cell growth under nutrient deprivation [5], to the calpain-mediated growth factor-induced cell proliferation, angiogenesis and cell migration [3, 6]. In addition, the proteolytic products of calpains have critical roles in a number of pathologies including cancer [3, 7–10]. Aberrant expression of *calpains* (*CAPN*s) has been reported in several types of cancer including colorectal carcinoma (CRC), one of the most prevalent types of cancer worldwide [9–11]. However, since the mechanisms of *CAPN* regulation/deregulation and its targets in pathological conditions are unknown, the prognosis value and benefits of the therapeutic inhibition of calpains cannot be definitely stablished. First, clinical data result controversial and mostly describe the aberrant expression of *CAPN*s, which is not necessarily related to an increase in enzymatic activity and substrate processing in tumor cells [9]. Second, although calpain-targeted strategies have been designed for therapeutic purposes [12], these proteases are also required for apoptosis induced by anticancer drugs. Thus, calpain unspecific inhibition can be both, beneficial and detrimental for tumor progression. These data bring about the important challenge of deciphering the mechanisms of spatial regulation and substrate recognition by specific calpains for the effective design of new anticancer therapeutic strategies.

As mentioned, substrate-recognition by calpains is a process poorly understood. In contrast to other proteolytic systems, calpain-mediated cleavage of proteins is not dependent on a previous post-translational modification such as ubiquitination of proteins to target them for proteasome degradation. In addition to a preference target sequence, calpains can also recognize secondary structures adjacent to the cleavage site in their substrates, a fact that further complicates the identification of new calpain-substrates [4, 13].

It has been recently suggested that the subcellular compartmentalization of calpains would limit their access to specific substrates [14–17]. Calpain-binding to phospholipids at cell membrane, drive cell migration through the cleavage of adhesion proteins [17, 18]. Asymmetrically distributed calpain-2 at the rear of the cell is involved in the Epidermal Growth Factor Receptor (EGFR)-induced locomotion of cultured fibroblasts [19]. Calpains localized to the intermembrane space of mitochondria, induce apoptosis by cleavage of apoptosis-inducing-factor (AIF) [20, 21]. The calpain system has also emerged as an important player in the lysosomal cell death pathway [21, 22]. During endoplasmic reticulum (ER) stress, calpain localized in ER proteolyzes and activates caspase-12, inducing cell death in a cytochrome c- and Apaf-1-independent manner [23]. Finally, calpains have also a role in the nuclear compartment inducing either cell death by destabilization of nuclear membrane, or signaling pathways through the modulation of transcription factor activity, histone H3 cleavage or topoisomerase I nucleolar localization [21, 24–26]. Therefore, from that point of view, the question would not be just the levels of *CAPN* expression or activity, but also where within the cell these proteases exhibit their activity.

In this study we explore the calpain recruitment to a specific cell compartment as a mechanism for substrate recognition and function in colorectal tumor cell lines. We describe a new localization for the ubiquitously expressed calpain-2 within nucleoli of tumor cells. Our findings strongly suggest a role for nucleolar calpain-2 as a sensor for growth-inducing factors, repressing ribosomal biogenesis when cells experience unfavorable growth conditions. Moreover, our results show that the calpain-2-mediated repression of rRNA abundance in serum-deprived CRC cells is dependent on KRAS mutational status.

## RESULTS

### Subcellular localization of calpain in colorectal cancer cells

**Figure 1:**
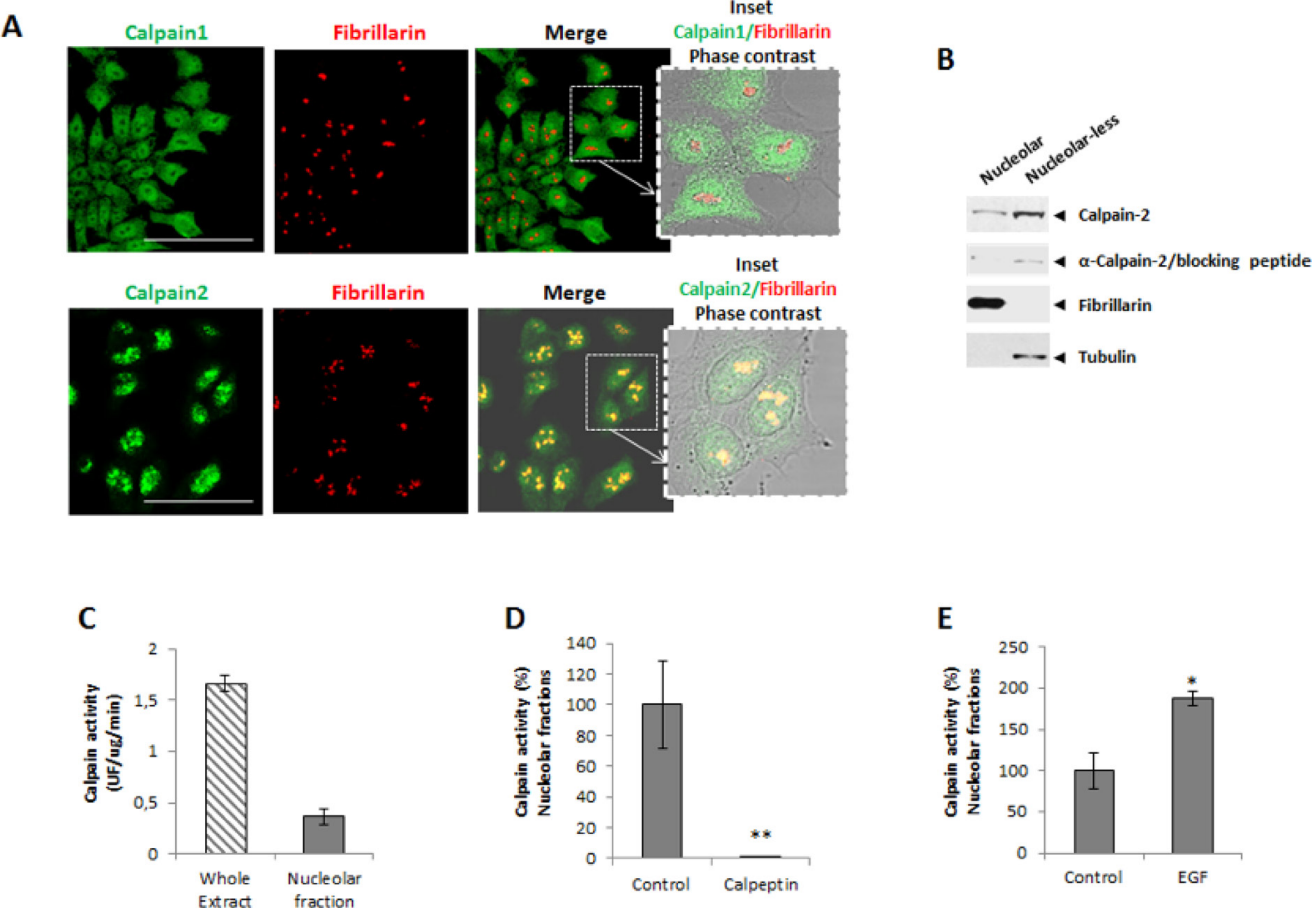
Subcellular localization of classical calpains in colorectal cancer DLD-1 cells (**A**) Immunofluorescence staining of calpain-1 and calpain-2 (green), fibrillarin (red) and merge in 24 h serum-starved cells. Inset shows merge images of immunofluorescent staining and phase contrast. Scale bars 75 μm. (**B**) Calpain-2 in nucleolar and nucleolar-less fractions analyzed by western blot. The specificity of the band recognized by the antibody was confirmed by the use of a blocking peptide with the same anti-calpain-2 antibody. Fibrillarin (nucleolar) and tubulin (nucleolar-less) were used as markers to assess the purity of subcellular fractions. (**C**) Calpain activity in whole cell extracts and nucleolar fractions. (D-E) Calpain activity in protein extracts from control or after 5 min treatment with calpeptin (**D**) or EGF (**E**). Values are shown as means ± SEM expressed as percentage of calpain activity vs. Control cells. ^*^p ≤ 0.05 and ^**^p ≤ 0.001.

Incubation of DLD-1 cells with a polyclonal antibody recognizing calpain-1 or -2 showed a marked immunofluorescence staining in nuclei. Calpain-1 staining although observed in the nuclear compartment was barely detected in nucleoli of DLD-1 cells (Figure 1A). Surprisingly, calpain-2 was strongly accumulated in nucleoli as evidenced by its colocalization with the nucleolar marker fibrillarin (Figure 1A). The same pattern of calpain-2 distribution was also observed in a human breast cancer cell line (Supplementary Figure 1) suggesting that the nucleolar calpain-2 localization was not cancer-type specific.

The presence of calpain-2 in nucleoli of DLD-1 cells was corroborated by western blot (Figure 1B) in nucleolar fractions and nucleolar-less fractions (comprising whole cell extracts excepting nucleoli). Experiments with a blocking peptide confirmed the specificity of the band recognized by the antibody.

We could hypothesize that the nucleolus is sequestering calpain-2 to limit its excessive activity in nuclei from DLD-1 cells. Consequently, the high concentration of calpain-2 found in nucleoli might favor its intermolecular autolysis and accordingly, the inactivation of calpain-2. A reduced but detectable calpain activity was found in nucleolar fractions when compared to the enzymatic activity from whole cell extracts (Figure 1C). This activity was not a residual calpain activity since it was sensitive to inhibition or stimulation with calpeptin and Epidermal Growth Factor (EGF), respectively. Calpain activity, while completely blocked in nucleolar fractions from calpeptin-treated cells (Figure 1D), was stimulated two fold after a 5 min treatment with EGF (Figure 1E). These data suggest that the nucleolar calapin is not a dead enzyme and therefore could recognize and process nucleolar substrates.

### Role of nucleolar calpain-2 in ribosomal biogenesis

**Figure 2:**
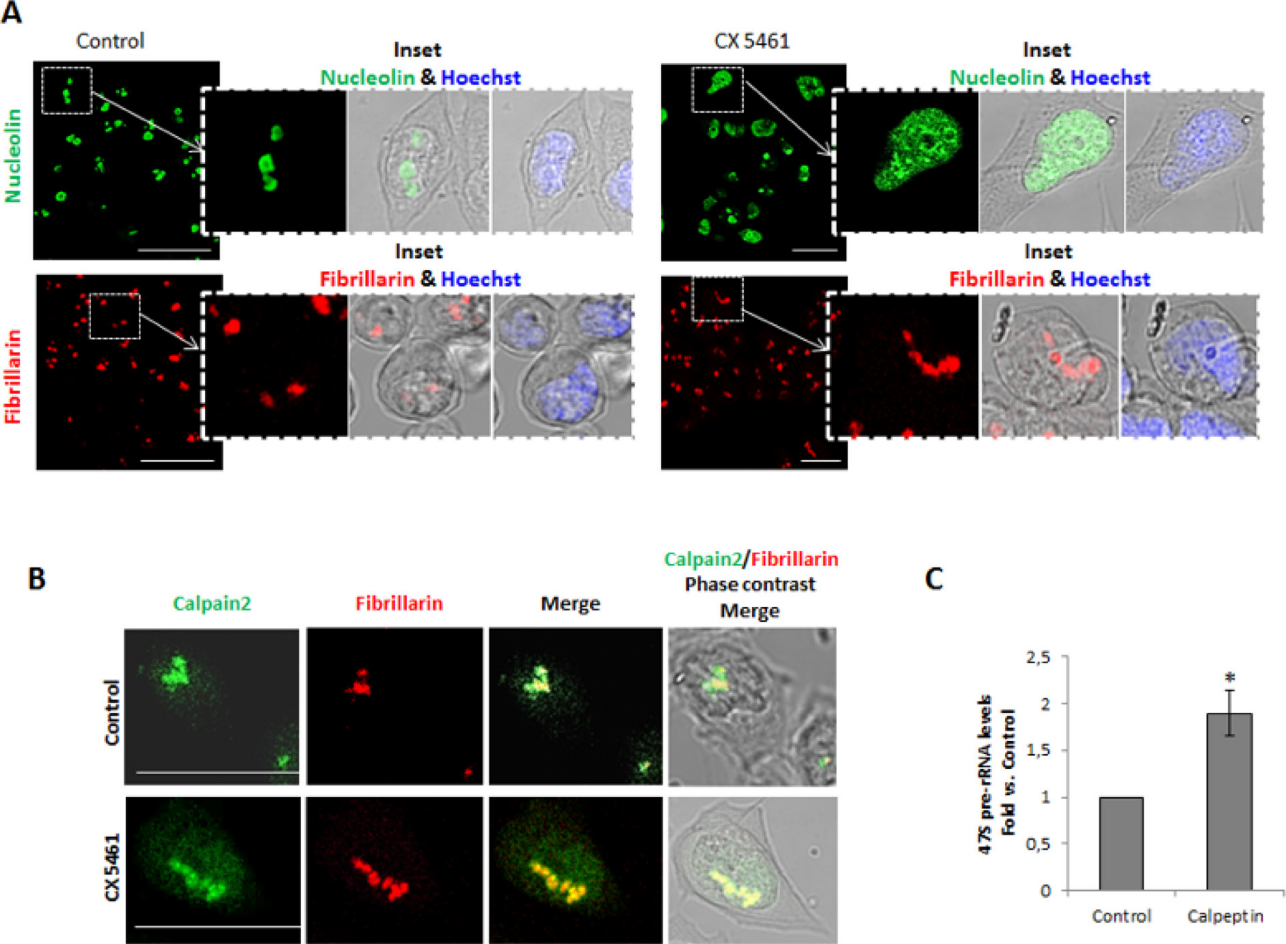
Calpain-2 relationship with rRNA biogenesis in DLD-1 cells (**A**) Nucleoli disassembly in control (left) and CX5461-treated (right) cells. The effectiveness of nucleoli disruption was assessed by immunofluorescent staining with anti-nucleolin antibody (green) and anti-fibrillarin (red). Scale bars 75 μm. Insets show zoom of merge images of nucleolin or fibrillarin with phase contrast. Merge of nuclear staining with Hoechst 33342 (blue) and phase contrast is also shown. (**B**) Association of calpain-2 with the nucleolar subcomponent fibrillarin during nucleoli disassembly. Immunofluorescence staining of calpain-2 (green), fibrillarin (red) and merge in control and CX-5461-treated cells. Merge images of immunofluorescent staining and phase contrast are shown. Scale bars 25 μm. (**C**) 47S pre-rRNA levels in control or calpeptin-treated cells measured by qPCR. DLD-1 cells were serum-starved for 24 h and further cultured for 24 h in the presence of vehicle (control) or calpeptin. RT-qPCR data are plotted as fold vs. control cells. Data (*n* ≥ 3) are mean ± SEM. ^*^p ≤ 0.05.

Since we found that calpain-2 is a functional protease in the nucleolar compartment, we asked whether it could have a role in ribosomal biogenesis. RNA Pol I inhibition causes the nucleoli to unravel into intranuclear structures thought to be single units of rDNA transcription retaining components of the transcriptional machinery such as Pol I and fibrillarin [27]. To investigate whether calpain-2 is actively involved in the ribosomal biogenesis in DLD-1 cells, its association with these nucleolar subcomponents during nucleoli disassembly was assessed. After CX5461 exposure (a specific inhibitor of Pol I), nucleoli were disrupted and some of their contents such as nucleolin, were largely spread over the nucleoplasm (Figure 2A upper panels). As already described [28], nucleolar substructures retaining fibrillarin immunoreactivity were also observed (Figure 2A lower panels). During CX5461-mediated nucleolar disassembly, calpain-2 colocalized with fibrillarin (Figure 2B), a protein from the dense fibrillary component (DFC) involved in both transcription and processing of pre-rRNA [28], indicating that calpain-2 has a dynamic relationship with rRNA biogenesis.

Calpain-2 could be either repressing or inducing rRNA accumulation. Inhibition of calpain activity by calpeptin in quiescent cells strongly increased 47S pre-rRNA levels (Figure 2C). We can conclude that calpain-2 is involved in the down-regulation of rRNA accumulation

### Nucleolar translocation of calpain-2

**Figure 3:**
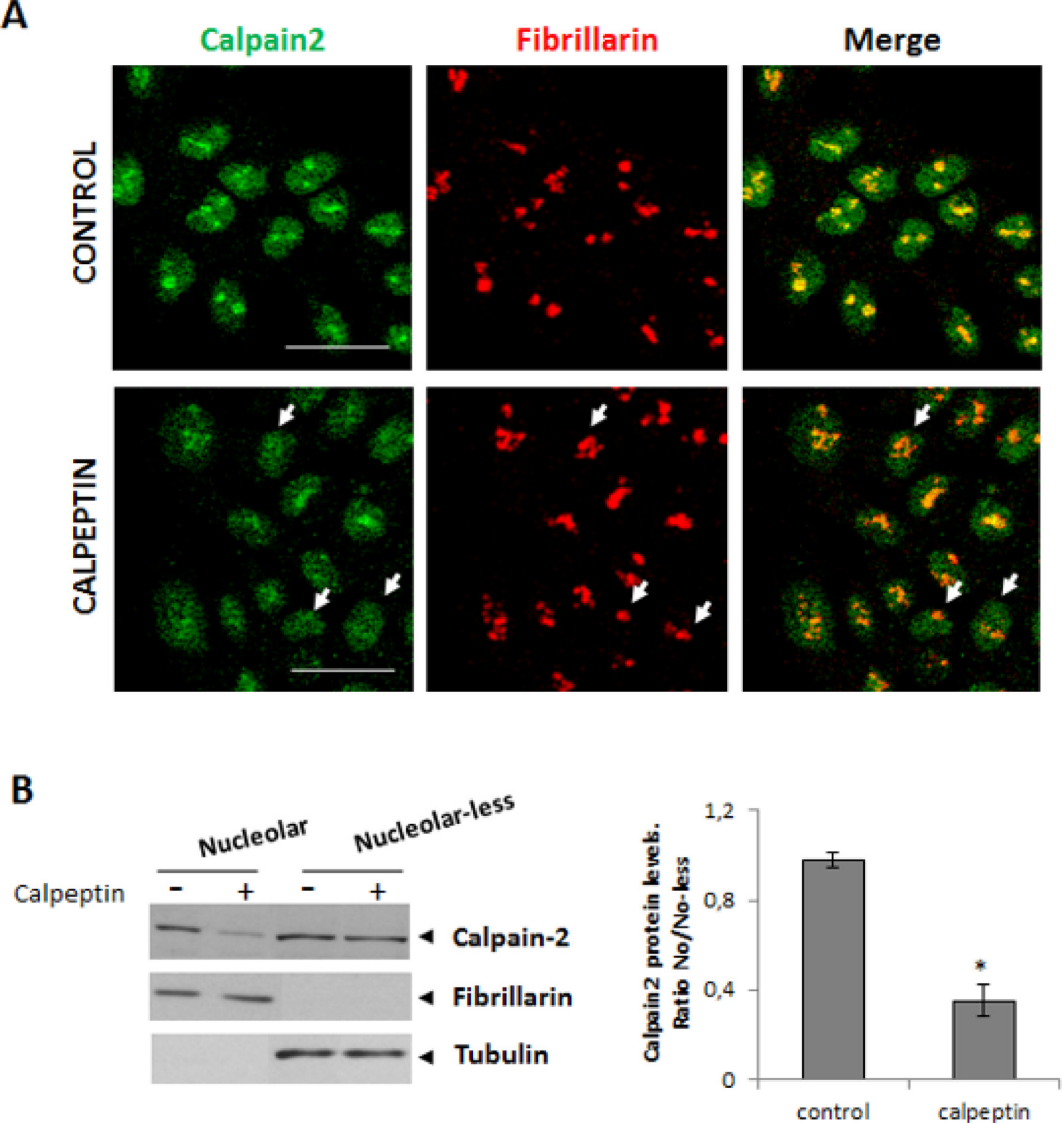
Localization of nucleolar calpain-2 after calpain activity inhibition DLD-1 cells were serum-starved for 24 h and further cultured for 24 h in the presence of vehicle (control) or calpeptin. (**A**) Immunofluorescent staining of calpain-2 (green), fibrillarin (red) and merge in control and calpeptin-treated DLD-1 cells. Scale bars 25 μm. Arrows point out to representative cells with low nucleolar calpain-2 and high fibrillarin staining in calpeptin-treated cells. Yellow and orange staining indicate a high and a poor co-localization of both proteins, respectively. (**B**) Western blot analysis of calpain-2 in nucleolar and nucleolar-less fractions from control and calpeptin-treated cells. Fibrillarin and α- tubuline were analyzed as markers of fraction purity. Proteins were quantified and normalized by their respective fraction markers. The ratio of nucleolar/nucleolar-less calpain-2 is represented as mean ± SEM. ^*^p ≤ 0.05 *vs*. control cells.

To determine whether calpain-2 accumulation into the nucleolar sub-structure was dependent on its enzymatic activity, the nucleolar localization of the enzyme was analyzed by immunofluorescence methods in quiescent 24 h serum-starved cells cultured in the presence or absence of calpeptin. As shown in Figure 3A, calpain-2 nucleolar localization was lost after inhibition of calpain activity in a vast number of cells. The western blot analysis confirmed that calpain-2 levels in the nucleolar compartment were dramatically reduced after calpeptin-treatment (Figure 3B). Conversely, no difference between control and calpeptin-treated cells was found in the rest of the cell (nucleolar-less fraction). No change in cell survival was observed after 24h-treatment with calpeptin (Supplementary Figure 2). From this data we should conclude that calpain activity is needed not only for the modulation of rRNA levels, but also for calpain-2 nucleolar accumulation.

### Signaling pathways involved in the nucleolar localization of calpain-2

The MAPK signaling pathway has been largely known to be involved in the modulation of calpain activity and subcellular distribution [18, 19]. The nucleolar activity associated to ribosomal biogenesis has also been linked to MAPKs pathways [29]. Since DLD-1 cells carry both, KRAS (G13D) and PI3K (E545K) activating mutations, we asked whether nucleolar calpain-2 localization was dependent on these pathways.

**Figure 4:**
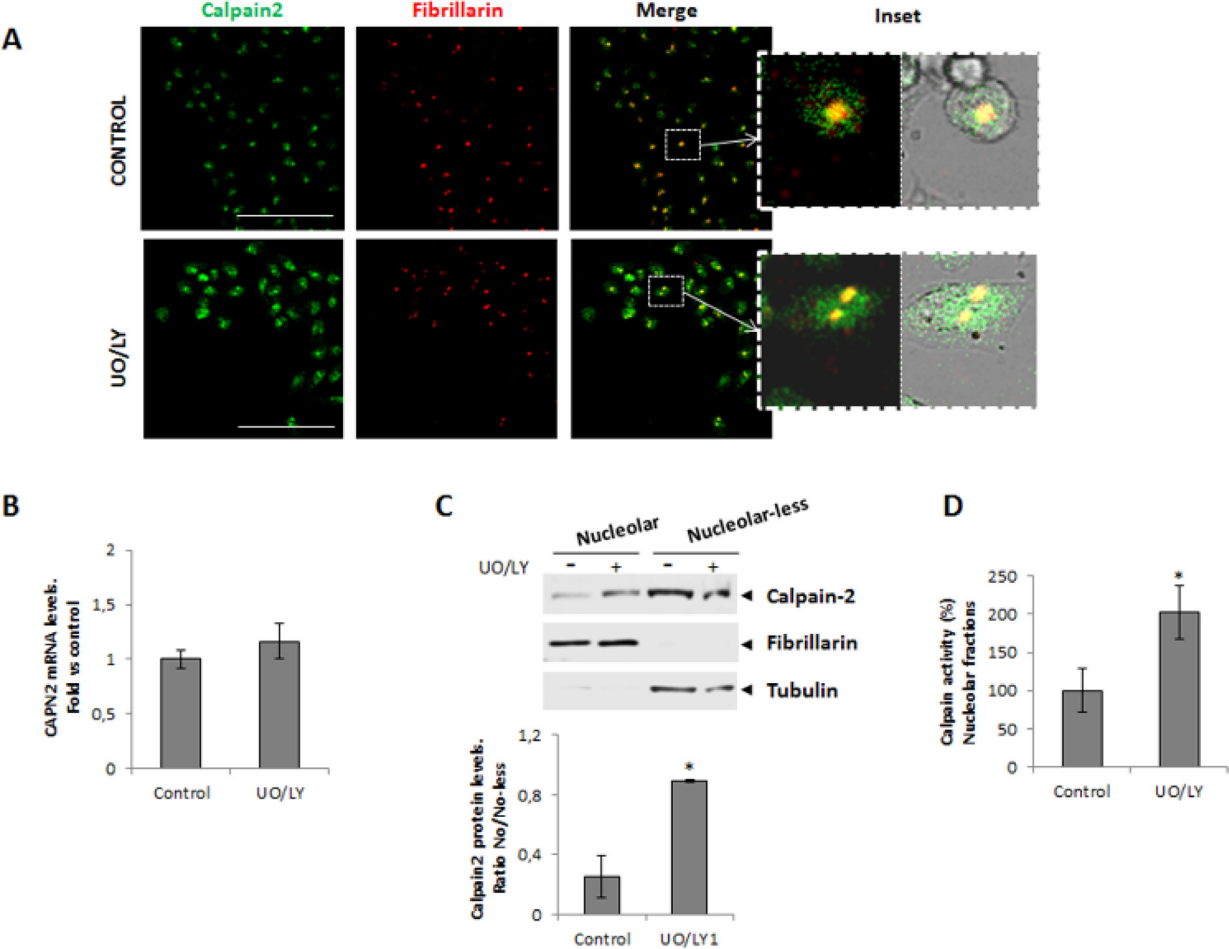
Role of MAPK/PI3K signaling pathway in the nucleolar localization of calpain-2 DLD-1 cells were serum-starved for 24 h and further cultured for 24 h in the presence of vehicle (control) or MEK and PI3K inhibitors (UO/LY). (**A**) Immunofluorescent staining of calpain-2 (green), fibrillarin (red) and merge in control and UO/LY-treated cells. Scale bars 75 μm. Insets show zoom of merge images of fluorescence staining and phase contrast. (**B**) *CAPN2* mRNA levels in control or UO/LY-treated cells analyzed by RT-qPCR. Data (*n* ≥ 3) are plotted as mean fold ± SEM *vs*. control cells. (**C**) Western blot of calpain-2 in nucleolar and nucleolar-less fractions from control and UO/LY-treated cells. Fibrillarin and tubulin were analyzed as markers of fraction purity. Proteins were quantified and normalized by their respective fraction markers. The ratio of nucleolar/nucleolar-less calpain-2 is represented as mean ± SEM. ^*^p ≤ 0.05 *vs*. control cells. (**D**) Calpain activity in nucleolar fractions from control or UO/LY-treated cells. Values (*n* ≥ 3) are mean ± SEM expressed as percentage of calpain activity *vs*. control cells. ^*^p ≤ 0.05.

Different lines of evidence support that tumors with both KRAS and PI3K mutations are unresponsive to the inhibition of MEK alone or the PI3K alone [30–33]. Dual targeted inhibition of both pathways is required for the effective blocking of mutated KRAS and PI3K downstream effects [33]. Consequently, to completely block these signaling pathways both inhibitors were simultaneously added to 24h serum-starved DLD-1 cells. Increased calpain-2 immunostaining was observed in both, the nuclear and nucleolar compartments after UO/LY-treatment (Figure 4A). This increase could be the result of either, induced *CAPN2* expression or protein redistribution. As shown, PI3K/MEK inhibitors had no effect on *CAPN2* mRNA levels (Figure 4B). However, the protein ratio of nucleolar/nucleolar-less calpain-2 dramatically increased in DLD-1 cells after PI3K/MEK inhibition (Figure 4C). Accordingly, calpain activity in nucleolar fractions was also induced after UO/LY treatment (Figure 4D). These data suggest that these signaling pathways are involved in the modulation of transport and accumulation of fully active calpain-2 in nucleoli of DLD-1 cells.

### KRAS-dependent localization of calpain-2 into nucleoli of CRC cells

Our previous experiments show that both, inducers (Figure 1E) and inhibitors (Figure 4) of KRAS/PI3K pathway, increase nucleolar calpain-2. Since DLD-1 cells harbor both, a wild type and a KRAS G13D mutated allele, is difficult to dissect the role of this pathway in the calpain-2 nucleolar import, retention or enzymatic activation. We examined those possible KRAS-dependent effects on calpain-2 nucleolar localization in two isogenic human CRC cell lines harboring the same PI3K activating mutation but with a different KRAS mutational status by deletion of either, the wild-type (DMUT) or the mutant allele (DWT7) found in *KRAS*G13D/WT DLD-1 cells.

**Figure 5:**
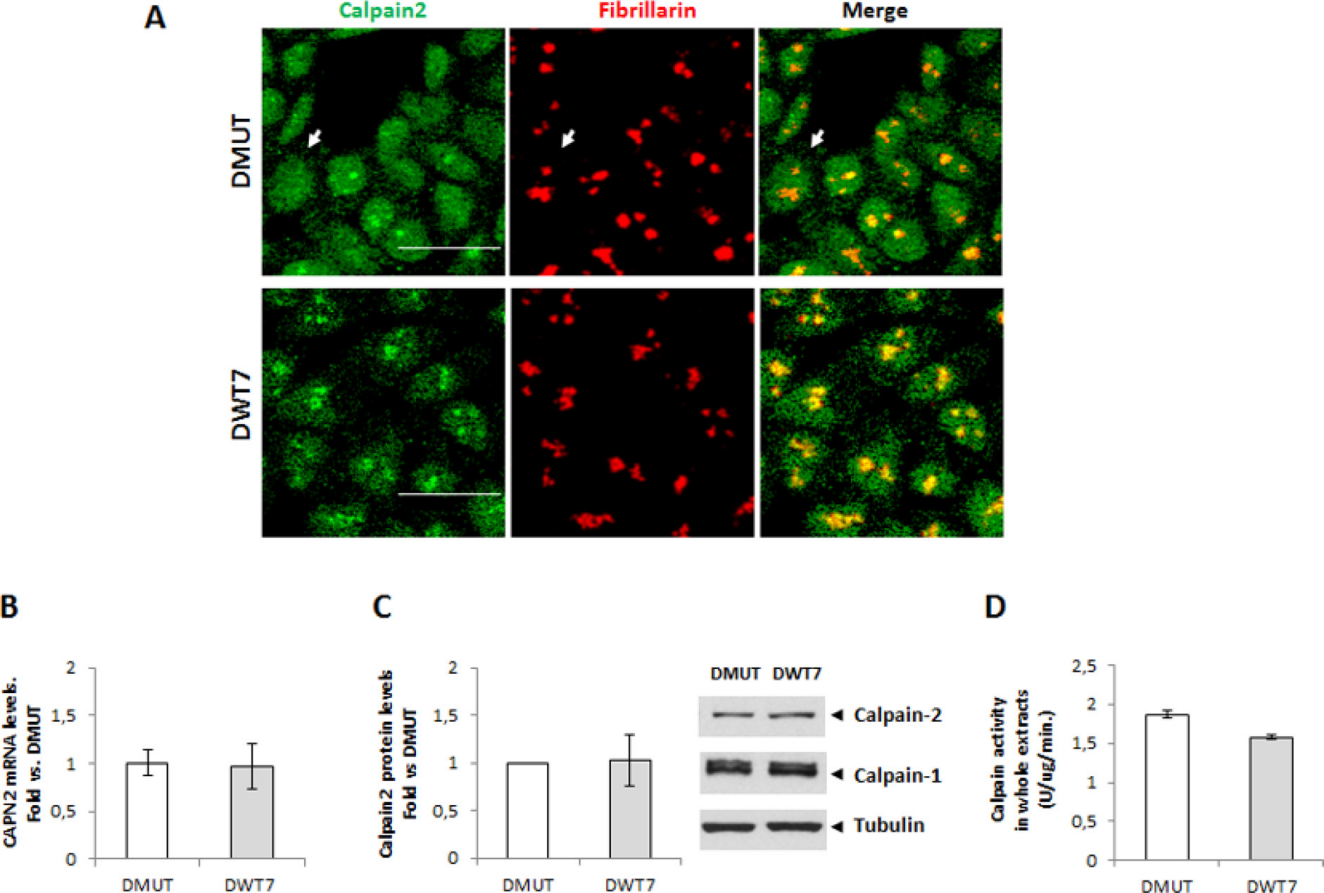
Effect of KRAS-mutational status on calpain-2 localization and expression in CRC cells (**A**) Immunofluorescent staining of calpain-2 (green) and fibrillarin (red) in two isogenic cell lines with different KRAS-mutational status, DMUT and DWT7. Merge images are shown. Scale bars 25 μm. Arrows point to a representative cell with no detectable nucleolar calpain-2 in DMUT cells. (**B**) *CAPN2* mRNA levels in DMUT and DWT7 cells were analyzed by RT-qPCR. (**C**) Total calpain-2 and calpain-1 protein levels in whole cell extracts from DMUT and DWT7 analyzed by western blot. Expression data were quantified and normalized against α-tubulin. (**D**) Total calpain activity in whole protein extracts from DMUT and DWT7 cells. Normalized data in (B) and (C) were plotted as fold *vs*. DMUT cells. Data (*n* ≥ 6) are mean ± SEM. No significant difference was found between cell lines.

Immunofluorescence staining of calpain-2 showed that the protease was mainly localized in nuclei of both DMUT *KRAS*G13D/- and DWT7 *KRAS*WT/-cell lines. However, a lower percentage of cells showing nucleolar accumulation of calpain-2 was observed in DMUT cell line (Figure 5A). In contrast, no difference in *CAPN2* gene expression, total protein levels, or enzymatic activity in whole cell extracts was observed between both cell lines (Figure 5B–5D).

**Figure 6:**
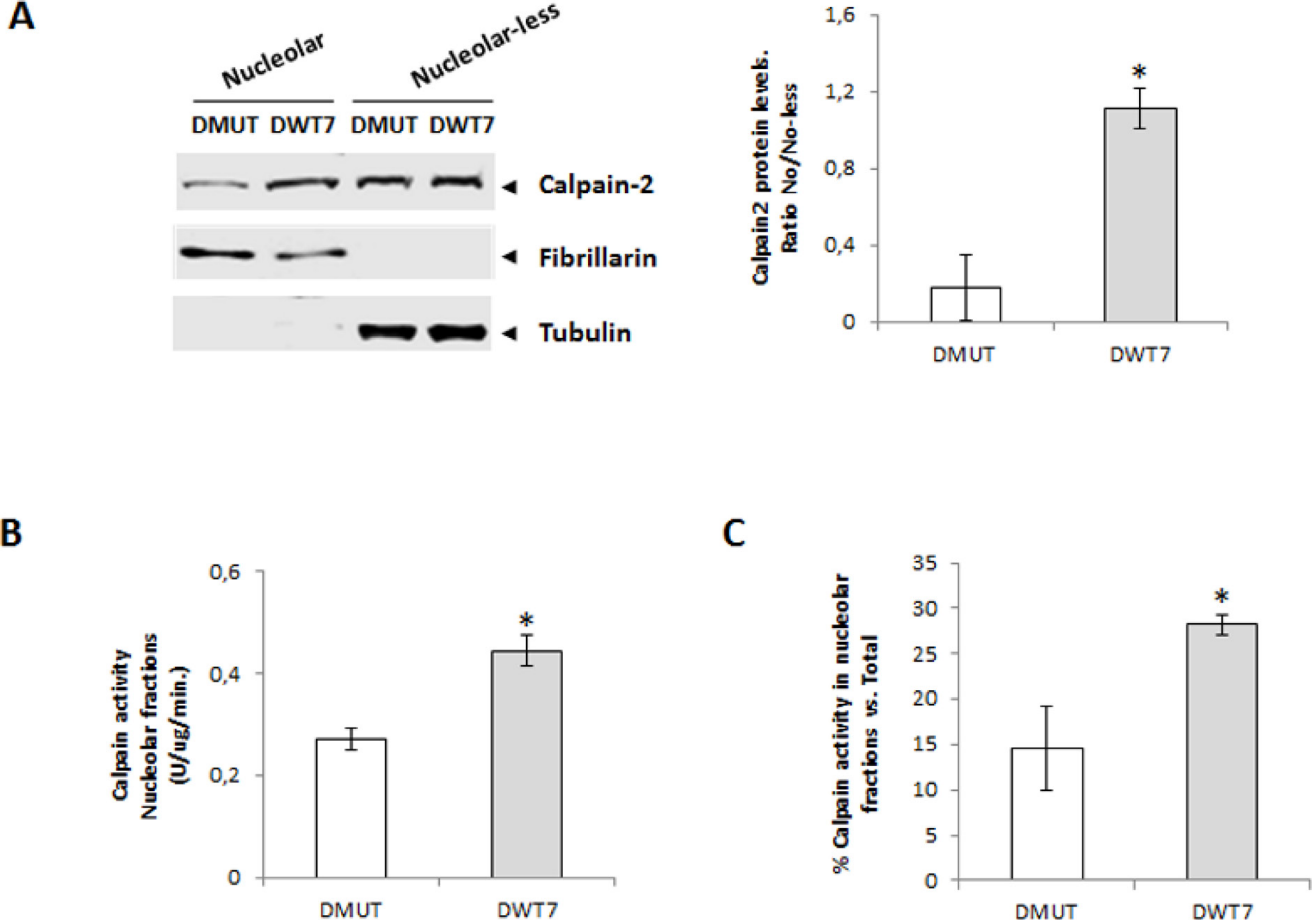
Nucleolar calpain-2 levels in CRC cell lines with different KRAS mutational status (**A**) Calpain-2 analyzed in DMUT and DWT7 cells by Western blot in nucleolar and nucleolar-less fractions. Fibrillarin and tubulin were used as markers of fraction purity. Proteins were quantified and normalized by their respective fraction markers. The ratio (*n* ≥ 6) of nucleolar/nucleolar-less calpain-2 is represented as mean ± SEM. ^*^p ≤ 0.05 *vs*. DMUT cells. (**B**) Calpain activity in nucleolar fractions from DMUT and DWT7 cells. Values (*n* ≥ 6) are mean ± SEM ^*^p ≤ 0.05. (**C**) Calpain activity in nucleolar fractions from DMUT and DWT7 cells. Values (*n* ≥ 6) are mean ± SEM expressed as percentage of nucleolar calapin activity from total activity in the cell. ^*^p ≤ 0.05.

Interestingly, in nucleolar fractions calpain-2 protein levels were higher in DWT7 than in DMUT cells (Figure 6A). Calpain activity not only was higher in nucleoli from DWT7 when compared with DMUT cells (Figure 6B), but in addition, it represented a higher percentage from total calpain activity found in the whole cell (Figure 6C). These data confirmed that the KRAS signaling pathway either directly or indirectly is involved in the modulation of calpain-2 nucleolar transport. Moreover, constitutively activated KRAS seems to be at least partially blocking nucleolar calpain-2 accumulation.

### Functional role of nucleolar calpain-2 in response to serum withdrawal

**Figure 7:**
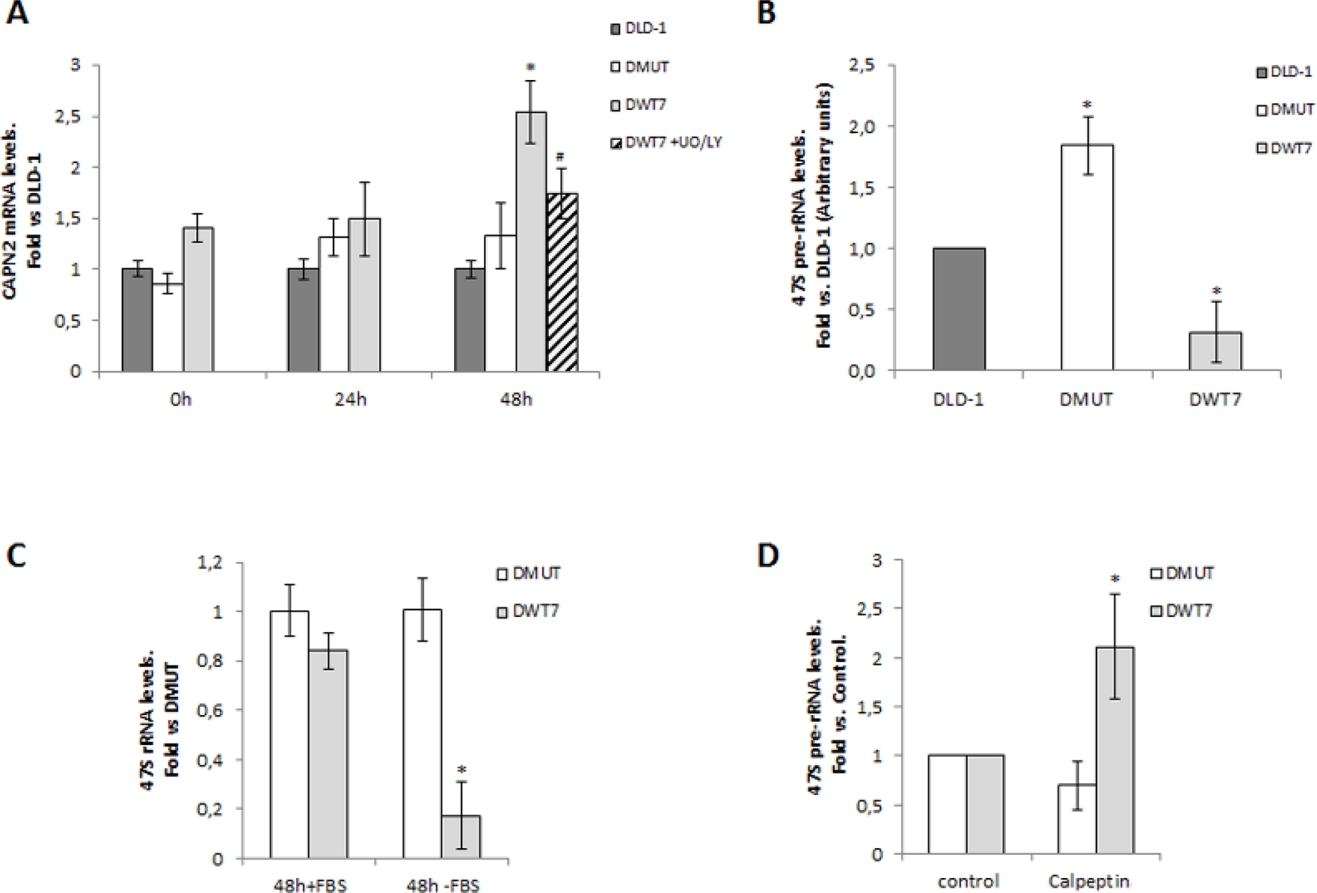
Correlation between calpain-2 expression and ribosomal biogenesis in response to serum-deprivation in CRC cell lines Cells were serum-starved for 0, 24, and 48 h. (**A**) *CAPN2* mRNA levels were analyzed by RT-qPCR at the indicated time points in DLD-1 (dark grey bars), DMUT (white bars) and DWT7 (light grey bars). DWT7 cells were also cultured for the last 24 h in the presence of UO/LY (dashed bars). Data (*n* ≥ 6) are expressed as fold *vs*. DLD-1 cells. ^*^p ≤ 0.05 compared with any time point in all the cell lines. ^#^*p* ≤ 0.05 compared with untreated 48 h-starved DWT7 cells. (**B**) 47S pre-rRNA levels after 48 h of serum deprivation were analyzed by RT-qPCR in the three cell lines. Data (*n* ≥ 9) are expressed as fold mean ± SEM *vs*. DLD-1. ^*^p ≤ 0.05 represents statistical difference compared to other cell lines. (**C**) 47S pre-rRNA levels were analyzed by RT-qPCR in DMUT and DWT7 cell lines cultured for 48 h in the presence (+FBS) or absence (-FBS) of serum. Data (*n* ≥ 3) are expressed as fold mean ± SEM. *vs*. DMUT (+FBS). ^*^p ≤ 0.01 shows significant difference compared to any group. (**D**) DMUT and DWT7 cells were serum-starved for 24 h and further cultured for 24 h in the presence of vehicle (control) or calpeptin. 47S pre-rRNA levels were analyzed by RT-qPCR in DMUT and DWT7 cell lines. qPCR data (*n* ≥ 5) are fold mean ± SEM, where ^*^p ≤ 0.05 fold *vs*. control.

Ribosomal biogenesis is tightly linked to cell growth in response to nutrient availability and growth signals. We hypothesize that consistent with a starvation-insensitive feature of tumor cells, constitutively activated KRAS could prevent nucleolar accumulation of calpain-2 and therefore ribosomal biogenesis might proceed even in the absence of serum. *CAPN2* mRNA levels were analyzed during the time course of a short-term serum starvation in CRC cell lines. As shown in Figure 7A, *CAPN2* mRNA levels increased at 48h starvation in DWT7. Conversely, in DMUT and DLD-1 cells *CAPN2* expression remained unchanged during the time course of the experiment.

Inhibition of PI3K/MEK prevented *CAPN2* upregulation in DWT7 cells (Figure 7A) but did not induce a significant change in the other cell lines (Supplementary Figure 3). Cell viability in DWT7 was not affected by UO/LY treatment (Supplementary Figure 4).

Interestingly, an inverse relationship between *CAPN2* mRNA and 47S pre-rRNA levels was found in CRC cell lines according to the KRAS mutational status (Figure 7B). 47S pre-rRNA levels were lower in DWT7, the cell line with the highest expression of *CAPN2* at 48h serum-starvation. The precursor rRNA was dramatically affected by the absence of serum in DWT7 cells (Figure 7C). Conversely, in agreement with our hypothesis of a starvation-insensitive DMUT cell line, 47S pre-rRNA levels remained unchanged along the experiment.

This data suggest a suppressor role for calpain-2 in ribosomal biogenesis in response to low nutrient and growth factors availability. Accordingly, 47S pre-rRNA levels increased in calpeptin-treated DWT7 cells (Figure 7D). As expected, 47S pre-rRNA levels in DMUT cells remained unchanged after calpeptin-treatment.

### Isoform-specific role of calpain-2 in rRNA synthesis

**Figure 8:**
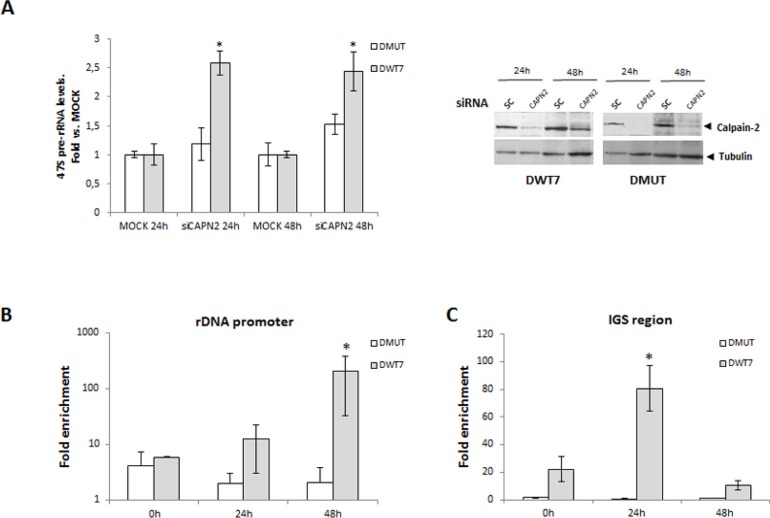
Isoform-specific function of calpain-2 in rRNA synthesis after serum deprivation (**A**) DMUT and DWT7 cells were transfected with MOCK or siRNA *CAPN2* and 24 h after transfection, cells were deprived of serum for 24 h and 48 h. 47s pre-rRNA levels were analyzed by RT-qPCR in knocked-down and MOCK cells at the indicated time points after serum-deprivation. Data (*n* ≥ 6) are shown as mean ± SEM fold *vs*. MOCK at the indicated time point for each cell line. ^*^p ≤ 0.05 *vs* MOCK. A representative western blot of calpain-2 in knocked-down and MOCK cells is shown. (B-C) Calpain-2 binding to rDNA core promoter (**B**) or to intergenic spacer (IGS) region (**C**) was analyzed by ChIP assay in DMUT and DWT7 samples during the time course of serum-starvation. qPCR data of calpain-2 bound to rDNA core promoter or IGS are represented as fold enrichment relative to the IgG control. The average of three independent experiments is shown (mean ± SEM), where ^*^p ≤ 0.01 *vs*. any group of samples in the experiment.

We have shown that calpain-2 is localized in nucleoli of CRC cells and that there is a correlation between *CAPN2* mRNA and 47S pre-rRNA levels, but other calpains could be involved in the modulation of ribosomal biogenesis. Calpeptin is a specific inhibitor of calpain activity, but it inhibits the activity of any calpain. To demonstrate the isoform-specific role of calpain-2 in pre-rRNA accumulation, 47S pre-rRNA levels were analyzed in *CAPN2* knockdown cells during the time course of serum starvation. 47S pre-rRNA levels were strongly increased when *CAPN2* was knocked-down in DWT7 cells, but remained unaffected in DMUT cells (Figure 8A). A high efficiency (≥ 80%) of siRNA-mediated depletion of calpain-2 was found in both cell lines (Figure 8A, right panel). These data indicates that calpain-2 is modulating the levels of 47S pre-rRNA and that this modulation is dependent on the mutational status of KRAS.

Reduced levels of 47S pre-rRNA could be the result of either, decreased rRNA synthesis or increased rate of pre-rRNA precursor cleavage and processing [28, 34]. To investigate the role of calpain-2 in rRNA synthesis, calpain-2 occupancy at several transcriptional regulatory regions of rDNA repeats was analyzed by chromatin immunoprecipitation (ChIP assay) during the time course of serum starvation. A time-dependent recruitment of calpain-2 to rDNA core promoter after serum starvation was only observed in DWT7 cells (Figure 8B). As shown in Figure 8C, a significant enrichment of calpain-2 at IGS region was found in DWT7 cells with a maximal peak at 24 h after serum depletion. Conversely, no change of calpain-2-binding to core promoter or IGS was observed in DMUT cells during the time course of the experiment. These data suggest a repressing function for calpain-2 in rRNA synthesis.

## DISCUSSION

Herein, we present evidences for a new subcellular localization of calpain-2 in nucleoli of CRC cells. Nucleolar CAPN3 localization has also been reported in melanoma cells [35]. Nevertheless, a number of studies have highlighted the role played by calpain-1 and -2 during malignant transformation [3, 5, 7–10, 12, 17]. However, the isoform-specific function of ubiquitously expressed calpains in CRC remains elusive [3, 7, 12]. A recent study with a reduced number of patients revealed that the activity and protein levels of calpain-2 were higher in colorectal adenocarcinoma than in normal colonic mucosa from the same patients [9]. Controversial data have been reported in other types of cancer [3, 7, 8]. In our studies no difference was found in total calpain-2 protein levels or enzymatic activity in whole cell fractions from CRC cell lines. However, important differences were observed when nucleolar calpain-2 was specifically studied in the different cell lines. We and others have previously hypothesized that the cell compartment would restrain the access of isoform-specific calpains to key substrates for the biological process they are involved in [14–26]. Consequently, calpain distribution is a critical step for their isoform-specific function.

An important function of nucleoli not related to ribosomal biogenesis is to immobilize selected proteins away from their downstream effectors or substrates in the nuclear compartment. A high concentration of calpain-2 *in vitro* has been shown to favor the intermolecular autolysis of the protease reducing its half-life [2]. Our data discard the intermolecular autolysis of calpain-2 in the nucleolus as a mechanism to reduce its half-life. Indeed, the apparent molecular weight of nucleolar calpain-2 analyzed by western blot corresponds to the full-length enzyme and the enzymatic activity analyzed in nucleolar fractions can be inhibited or induced. In addition, in the presence of calpeptin, the hypothetical intermolecular autolysis of calpain-2 should be inhibited resulting in higher levels of nucleolar calpain-2, but instead we found lower levels of nucleolar calpain-2 in calpeptin-treated DLD-1 cells. All this data demonstrate that nucleolar calpain-2 is a functional enzyme. Moreover, our results suggest that the full-active calpain-2 is needed for both, its nucleolar-localization and for its function in ribosomal biogenesis. The levels of calpain-2 in nucleolar fractions were strongly reduced and, repression of pre-rRNA synthesis was prevented when calpain activity was inhibited by its pharmacological inhibitor in DLD-1 cells.

We propose that nucleolar calpain-2 takes part of the program that tightly links environmental conditions (such as growing-factors found in serum) to ribosomal biogenesis. Transcription of rDNA genes in response to cell growth and proliferation is regulated by a variety of signaling pathways including MAPK/PI3K [29, 36]. Constitutive activation of these pathways during carcinogenesis has been shown to upregulate rRNA synthesis, a key step in tumorigenesis initiation [29]. Our data suggest that nucleolar calpain-2 might be a sensor for external cues, repressing rDNA transcription when cells are under adverse growth conditions. It has been shown that the wild-type KRAS allele can act as a tumor suppressor exhibiting a full activated signaling pathway, and triggering potent antitumor responses [31, 37]. Accordingly, our data indicate that in DWT7 cells harboring a single KRAS^WT^-allele nucleolar calpain-2 is involved in the modulation of pre-rRNA repression in response to serum-deprivation: (i) *CAPN2* mRNA levels are induced while pre-rRNA accumulation is strongly down-regulated after 48h serum-removal. Inhibition of calpain activity or depletion of calpain-2 by specific siRNA prevents this pre-rRNA down-regulation. (ii) Calpain-2 is recruited to rDNA promoter and IGS regions with a concomitant reduction of pre-rRNA levels. At this point, is noteworthy to mention that IGS sequences between rDNA transcription units contain several Pol I responsive promoters. Further supporting the role of nucleolar calpain-2 as a repressor of rRNA synthesis, intergenic transcripts are recognized as non-coding RNAs involved in the epigenetic transcriptional down-regulation of rRNA in response to stress, nutrient and growth factor restriction [34, 36].

Tumor cells acquire self-sufficiency and independency of extracellular signaling events through the constitutive activation of downstream kinases. A dominant mutated KRAS allele, promotes tumor progression by limiting the efficacy of MAPK signaling [37]. KRAS mutations render an activated form of RAS protein which is continuously signaling to downstream effectors, even in the absence of extracellular stimuli and therefore ribosomal biogenesis might proceed in the absence of nutrients or growing-inducing factors. Consistent with a starvation-insensitive feature of tumor cells with constitutively activated *KRAS*G13D/- , we observed that in DMUT cells the induction of *CAPN2* mRNA, the nucleolar accumulation of the protease and its binding to transcriptional regulatory elements on rDNA after serum-deprivation is prevented. Accordingly, pre-rRNA levels in DMUT cells remain unchanged when cells are cultured in the absence of serum.

Finally, the mechanism underlying calpain-2 function in ribosomal biogenesis or in the modulation of other nucleolar targets should be further studied. A limitation of most studies on the role of calpains in different biological processes is that their final output will depend on the calpain-isoform, subcellular distribution and substrates recognized [6, 7, 14–26]. Therefore, to dissect the role and interplay of organelle-specific calpain-2 it will be crucial to first elucidate the mechanisms of calpain-2 subcellular distribution. We can speculate with the mechanisms of calpain-2 nucleolar translocation. Calpain-2 has no recognized nucleolar localization signals (NOLSs) for the nucleolar import, but proteins lacking NOLSs can dimerize with NOLS-containing proteins to enter into the nucleolus [38]. Thus, it is reasonable to think that calpain-2 is most probably dimerizing with a NOLS-protein for nucleolar import. In addition, we cannot rule out the possibility of nucleolar calpain-2 accumulation as a result of increased nucleolar retention. Our results show that calpain activity seems to be needed for calpain-2 nucleolar localization. Thus, dimerization of calpain-2 with a NOLS-containing protein and/or nucleolar import/export might be dependent on its enzymatic activity. A relevant task will be the identification of such calpain-2-partners. Preventing nucleolar calpain-2 translocation would bring important insights into the precise role of this calpain, not only in the control of rRNA synthesis but also on other nucleolar targets and biological processes deregulated in cancer cells.

Several anti-cancer drugs have been designed to inhibit rRNA transcription, although not with the desired selectivity towards tumor cells [29]. Our findings open a new field of research in the modulation of rDNA transcription and cell growth associated to the mutational status of KRAS frequently found in CRC [11]. Understanding the mechanisms of calpain-2 nucleolar translocation will be a challenging task for the design of targeted-strategies to selectively inhibit calpain-2 nucleolar pathway in rapidly proliferating CRC cells.

## MATERIALS AND METHODS

### Materials

Primary antibodies against calpain-1 (ab39170), Nucleolin (ab22758) and α-Tubulin (ab52866) were purchased from Abcam. Other antibodies used were: α-Calpain-2 (Cell Signaling), α-Fibrillarin (Novus Biologicals), Alexa Fluor 488 anti-rabbit IgG (Invitrogen), and Cy3 anti-mouse (Sigma). Inhibitors of PI3K (LY294002) and RNA Pol I (CX5461) were both from Calbiochem. Inhibitors of MEK (UO126) and calpain activity (calpeptin) were purchased from Promega and Sigma, respectively. Epidermal Growth Factor (EGF) was obtained from R&D Systems.

### Cell culture

Human colon cancer cell lines DLD-1 (PI3K^E545K/WT^; KRAS^G13D/WT^) and their isogenic derivatives DMUT (PI3K^E545K/WT^; KRAS^G13D/−^) and DWT7 (PI3K^E545K/WT^; KRAS^-/WT^) were commercially acquired from the GRCF Biorepository and Cell Center, at Johns Hopkins University. Mutations in CRC were confirmed by the use of both, OncoGenBasic S1 & S1.v2 and S2 &S2.v2 kits for detection of somatic mutations Seqplexing (Sequencing Multiplex, Genetest). All cell lines were cultured in McCoy’s 5A Modified Medium (Gibco) supplemented with 10% fetal bovine serum (FBS), penicillin/streptomycin and (2mmol/L) L-glutamine and cultured under standard conditions in a humidified atmosphere at 37°C and 5% CO2.

For experiments, cells were plated in complete medium supplemented with 10% FBS and cultured during 24h; culture medium was then replaced by serum-free medium and cells further cultured for the indicated periods of time in the presence or absence of CX5461 (1 μM), Calpeptin (15 μM), U0126 (1 μM), LY294002 (10 μM) and EGF (100 ng/ml).

### Immunofluorescence analysis

CRC cells were cultured onto 13 mm Ø borosillicate Cover Glass (VWR 631-0149) and immunostained as described previously [17]. Briefly, cells were incubated with the indicated primary antibodies overnight at 4°C. The proper secondary antibody was used for detection. Nuclei were counterstained with Hoechst 33342 (Invitrogen). Pictures were acquired on a Leica TCS-SP 2 confocal microscope.

### Nucleoli isolation

Cells (3 × 10^6^) were platted on T225cm^2^ flasks and cultured as indicated above. Nucleoli isolation was performed as published [39]. Briefly, cells were washed 3 times and suspended in 1.5 ml cold Solution I (0.5 M sucrose, 3 mM MgCl_2_ with protease and phosphatase inhibitors). Next, cells were sonicated on ice at 40% amplitude, 10 s on, 10 s off, 10 times (Sonics, VCX130). The sonicated cells were checked under a microscope, in order to ensure that more than 90% of both, cells and nuclei were broken and the nucleoli released. In this method no nuclear fraction was obtained. Then, the cell lysate containing nucleoli and nuclear/cytosolic proteins was laid on 1.5 ml of Solution II (1.0 M sucrose, 3 mM MgCl_2_) and centrifuged at 1800 ×*g* for 10min at 4°C. The pellet was transferred to a new tube and labelled as *nucleolar* fraction. The supernatant was carefully removed and centrifuge again at 1800 × *g* for 10min at 4°C. This supernatant was labelled as *nucleolar-less* fraction containing whole cell extracts (cytoplasmic/nuclear proteins) excepting nucleoli. Fibrillarin was used as a marker to assess the purity and whole recovery of nucleoli in nucleolar fractions. Analysis of tubulin, a protein known to be found in both the nuclear and cytosolic compartments but not in nucleoli [40], was also used to discard contamination between fractions.

### Protein extraction and Immunoblotting

Total protein was extracted in RIPA buffer in the presence of protease inhibitors. Equal amounts of protein (20 *μ*g) were size-fractionated by SDS-PAGE gel electrophoresis and electroblotted onto nitrocellulose membranes (Protran^®^, Whatman). The specific proteins were detected using the indicated primary antibodies and HRP-conjugated secondary antibody. Blots were developed by enhanced chemiluminescence reaction (ECL Detection Kit, GE Healthcare). Equal loading or fraction purity was confirmed by reprobing the blot against α-tubulin or fibrillarin and by Ponceau Red staining.

### Calpain activity

Calpain activity was measured using a “calpain activity assay kit” (Calbiochem) according to the manufacturer’s instructions. Briefly, whole cells or nucleoli from cancer cell lines were solubilized in cell lysis buffer (CytoBuster™ Protein Extraction Reagent). Samples (in the presence or absence of inhibition buffer containing BAPTA) and standards, were then incubated during 15 min in a 96-well plate with activation buffer (containing Ca^2+^ and TCEP reducing agent) and the substrate (Suc Leu-Leu-Val-Tyr-AMC) provided in the kit. Fluorescence was measured using a fluorescence plate reader at an excitation wavelength of ~360–380 nm and an emission wavelength of ~440–460 nm. Calpain activity was determined as the difference between the activity obtained using the CAPN-inhibition buffer (BAPTA) and that detected with the activation buffer.

### RNA isolation and real time RT-qPCR analysis

Total RNA from CRC cell lines was extracted by RNAeasy Mini Kit (Quiagen), followed by treatment with DNase I for 10 min (RNase-Free DNase set, Quiagen). RNA was cleaned up by RNAeasy Mini Kit (Qiagen) and subsequently quantified using the NanoDrop ND-2000 (NanoDrop Technologies). RNA (1μg for *CAPN2* and 50 ng for 47S pre-rRNA) was reverse-transcribed to cDNA using a high-capacity RNA-to-cDNA kit (Applied Biosystems). cDNA products were amplified by qPCR using the GeneAmp Fast PCR Master Mix (Applied Biosystems) for *CAPN2* or Sybr Green PCR Master Mix (Applied Biosystems) for 47S pre-rRNA amplification. All reactions were carried out in triplicate. Quantitative real-time PCR was run in the 7900HT Fast Real-Time PCR System. *CAPN2* and 47S pre-rRNA were normalized according to 18S and actin quantification, respectively. Specific primers for *CAPN2* (Taqman Hs00965097_m1), 18S (Taqman 4319413E), 47S pre-rRNA (Fw 5’- GAACGGTGGTGTGTCGTTC-3’ and Rev 5’-GCGTCTCGTCTCGTCTCACT-3’) and *Actin* (Fw 5’-GTGCTATCCCTGTACGCCTC-3’ and rev 5’-GAGGGCATACCCCTCGTAGA3’) were purchased from Applied Biosystems. The threshold cycle (Ct) was determined, and the relative gene expression was expressed as follows: Relative amount , where *C*_t_ = *C*_t_ (*target*) – *C*_t_ (*housekeeping gene*) and ∆(∆*C*_t_)=∆*C*_t_ (sample)− ∆*C*_t_ (control).

### Calpain-2 knockdown by esiRNA

Cells were transiently transfected with 30nM *CAPN2* esiRNA (EHU025391-50UG), or Universal Negative Control #1 siRNA (SIC001), all of them purchased from Sigma. Cells were reverse transfected with Lipofectamine RNAiMAX (Life Technologies). The transfection reaction was carried out for 24 h. Dilutions of esiRNA and Lipofectamine where performed in Opti-MEM following manufacturer’s instructions. Cells were serum starved at 24 h after esiRNA transfection and collected 24 h and 48 h later to analyze the effects by western blot and qPCR.

### Chromatin immunoprecipitation

Cells were platted and cultured under standard conditions for 24h and harvested and crosslinked at 0, 24 and 48h after serum-deprivation. Fixed-cells were homogenized and nuclei and chromatin extracted as described elsewhere. Crosslinked chromatin was shared to 500bp average size by sonication (Vibra-Cell VCX-500 sonicator). A volume of 50μl of Protein A/G magnetic beads (Novex, Life technologies) in 1X PBS /1mg/ml BSA was incubated overnight with α-calpain-2 (IP) or rabbit IgG (MOCK) antibodies at 4°C. Equal amount of chromatin was immunoprecipitated with the corresponding antibody during 2 h. Immunocomplexes were recovered as previously described. 1% of starting chromatin was used as input to assure equal loading of chromatin for each sample. Purified DNA from Input, IP and MOCK samples was analyzed by qPCR using the Fast SYBR Green Master Mix (Applied Biosystems) following the manufacturer instructions with specific primers for human rDNA gene promoter (Fw 5›-GCCCCGGGGGAGGTAT -3› and Rev 5›-GAGGACAGCGTGTCAGCAATAA-3›), and intergenic spacer region IGS_36_ (Fw 5’- CGGGCCTTGGCAGATTC-3’ and Rev 5’- CG CGCGTAGAGGAGAGATTT-3’) [41]. Results were expressed as fold enrichment for calpain-2 on selected regions where ChIP signal is represented as the fold increase in IP signal relative to the background MOCK signal.

### Statistics

Data are presented as mean ± S.E.M. Statistical significance was estimated with one-sample Student’s *t*-test. Differences were considered significant at least at *p* ≤ 0.05. Independent experiments were conducted with a minimum of three replicates per condition to allow statistical comparison.

M.T-F and L.R-F are funded by Consellería de Educación [GVPROMETEO 2013-005 and GVPROMETEO 2014/II-055, respectively].

## SUPPLEMENTARY MATERIALS FIGURES



## Abbreviations

CRC: colorectal cancer
PI3K: Phosphatidylinositol-4, 5-bisphosphate 3-kinase
MAPK: Mitogen-activated protein kinase
CAPN: Calpain
EGF: Epidermal Growth Factor
DFC: Dense Fibrillar Component
IGS: Intergenic Spacer
NOLS: Nucleolar Localization Signal.
