# Correction: New localization and function of calpain-2 in nucleoli of colorectal cancer cells in ribosomal biogenesis: effect of KRAS status

**DOI:** 10.18632/oncotarget.25142

**Published:** 2018-04-06

**Authors:** Marcelino Telechea-Fernández, Lucia Rodríguez-Fernández, Concha García, Rosa Zaragozá, Juan R. Viña, Andrés Cervantes, Elena R. García-Trevijano

**Affiliations:** ^1^ CIBERONC, Department of Medical Oncology, INCLIVA Biomedical Research Institute/University of Valencia, Valencia, Spain; ^2^ Department of Biochemistry and Molecular Biology, INCLIVA Biomedical Research Institute/University of Valencia, Valencia, Spain; ^3^ Department of Anatomy and Human Embriology, INCLIVA Biomedical Research Institute/University of Valencia, Valencia, Spain

**This article has been corrected:** The correct author name is given below:

**Juan R. Viña**

Original article: Oncotarget. 2018; 9:9100-9113. https://doi.org/10.18632/oncotarget.23888

