# Supplementary material for: New localization and function of calpain-2 in nucleoli of colorectal cancer cells in ribosomal biogenesis: effect of KRAS status
Source: Oncotarget. 2018 Jan 3;9(10):9100–13. doi: 10.18632/oncotarget.23888 (PMC5823616; doi:10.18632/oncotarget.23888)
Supplement: Supplementary file 1 [file oncotarget-09-9100-s001.pdf]

New localization and function of calpain-2 in nucleoli of colorectal cancer cells in ribosomal biogenesis: Effect of KRAS status

SUPPLEMENTARY MATERIALS

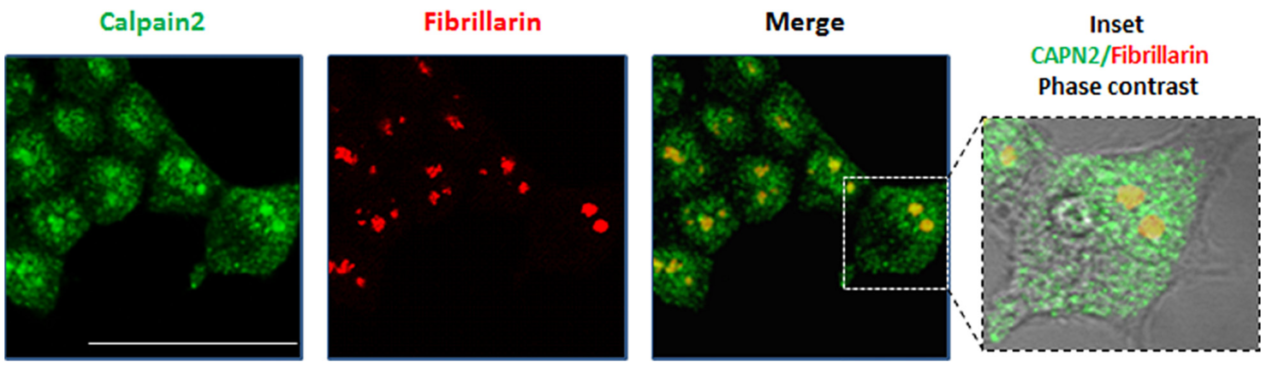

Supplementary Figure 1: Subcellular distribution of calpain-2 in MCF-7 breast cancer cell line. Immunofluorescence staining of calpain-2 (green), fibrillarin (red) and merge in 24h serum-starved cells. Inset shows merge images of immunofluorescent staining and phase contrast. Scale bars 25 µm.

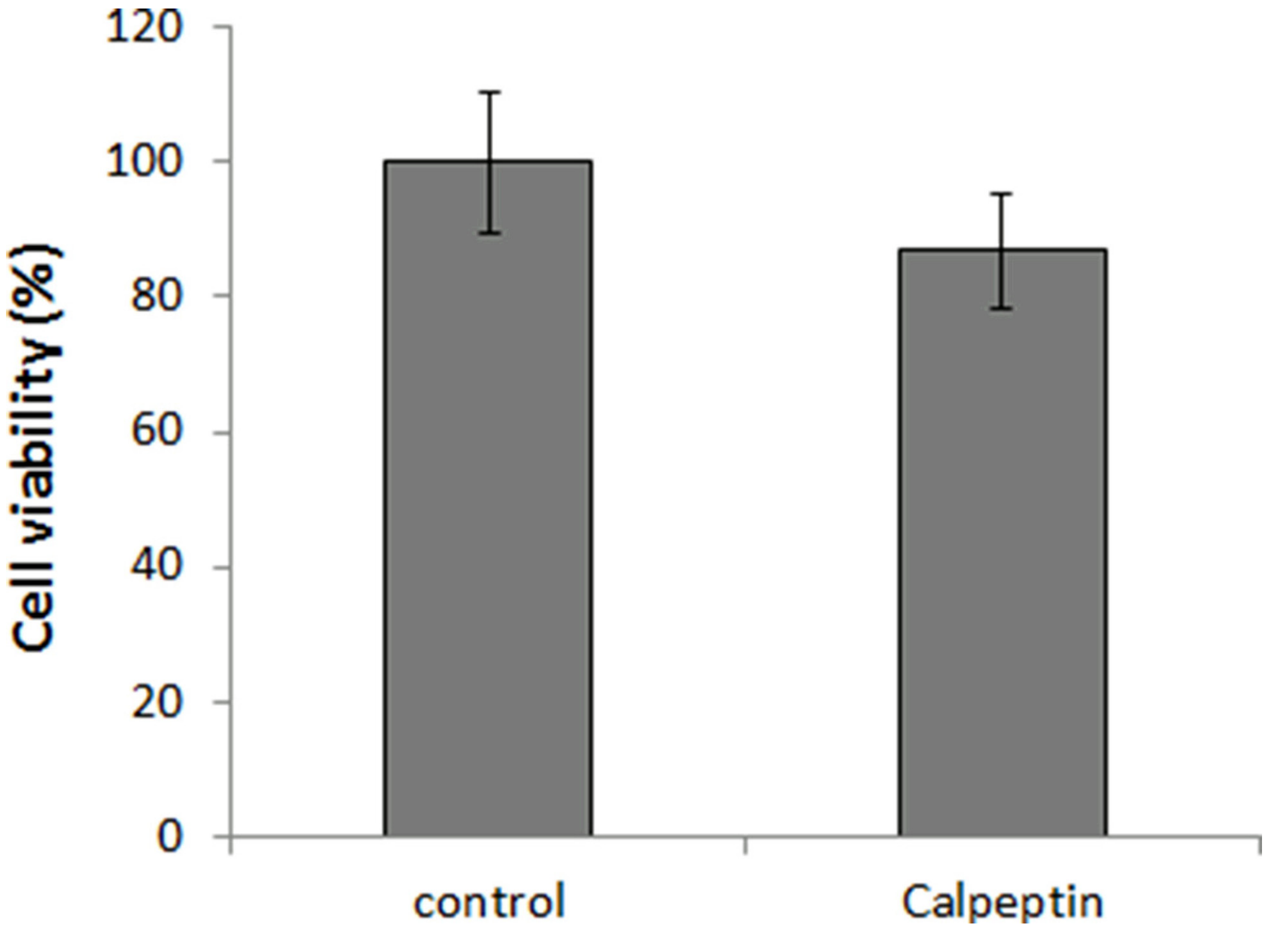

Supplementary Figure 2: Cell viability in calpeptin-treated DLD-1 cells. 48h serum-starved cells were cultured for the last 24h in the absence or presence of calpeptin. MTT assay was performed to analyze cell viability. Values (*n* = 3) are shown as means ± SEM expressed as percentage of cell viability vs. Control cells. No statistical difference was found.

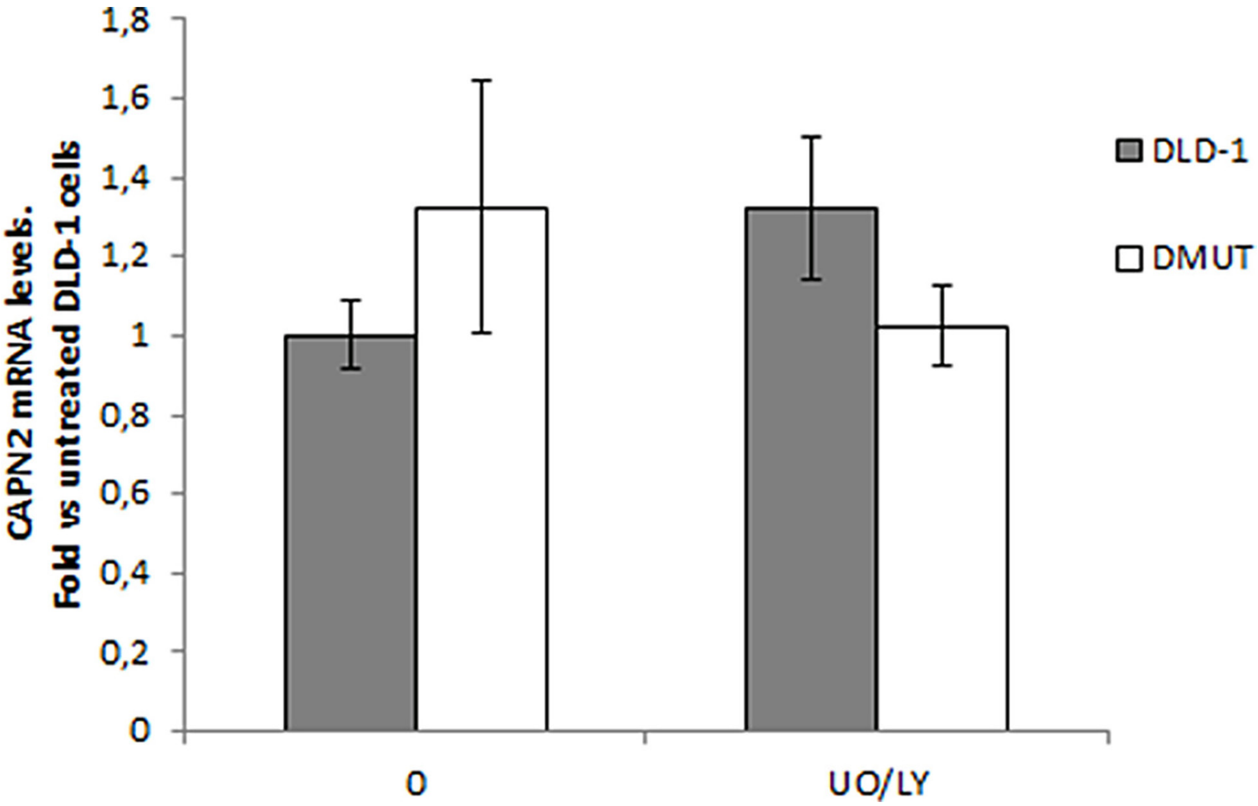

Supplementary Figure 3: Calpain-2 expression in DMUT and DLD-1 after UO/LY-treatment. 48h serum-starved cells were cultured for the last 24h in the absence (control) or presence of MAPK/PI3K inhibitors (UO/LY). CAPN2 mRNA levels were analyzed by RT-qPCR in DLD-1 (grey bars) and DMUT (white bars) cells. Data (n³6) are means ± SEM expressed as fold vs. DLD-1 control cells. No statistical difference was found.

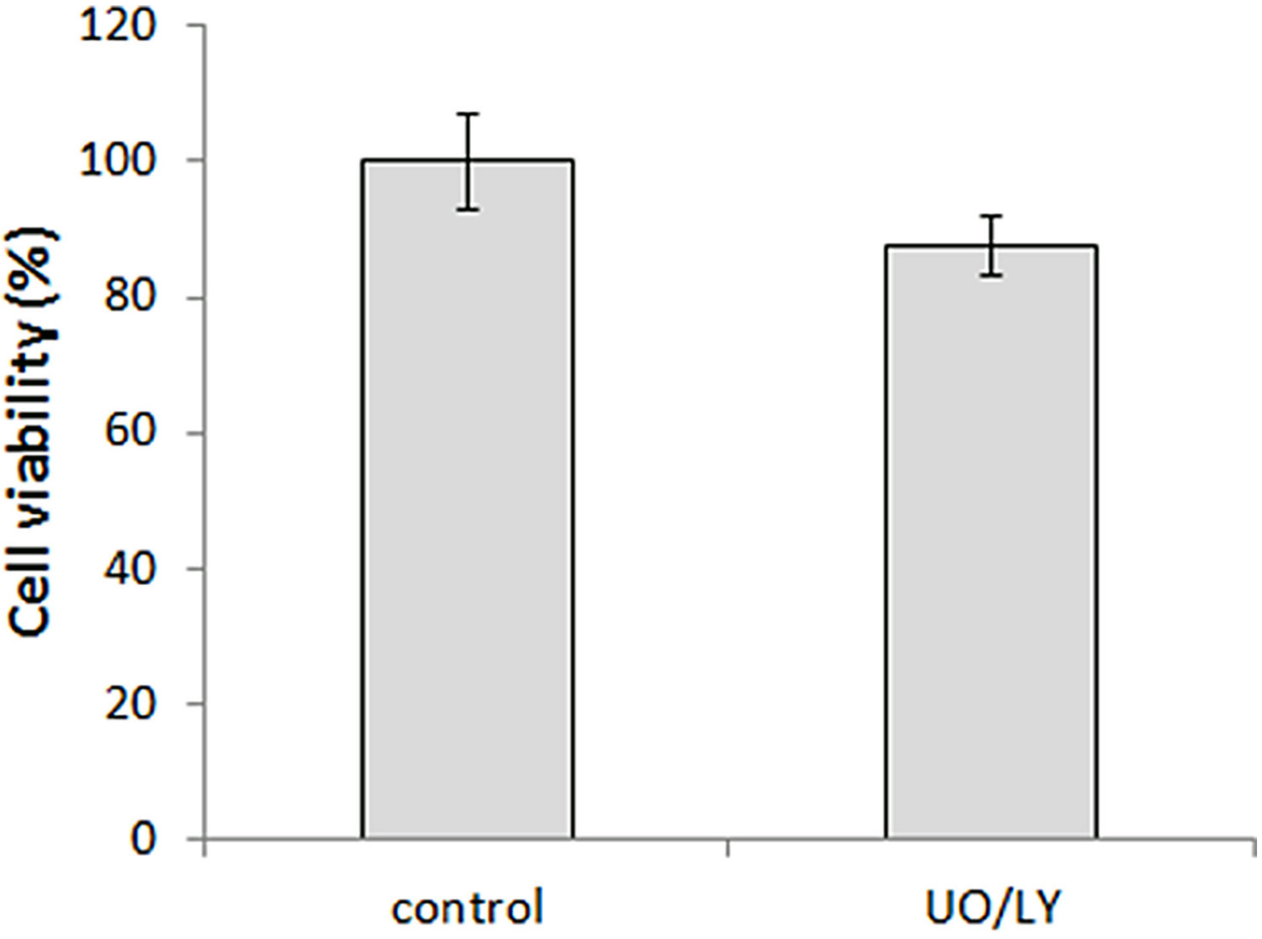

Supplementary Figure 4: Cell viability in UO/LY-treated DWT7 cell line. Cells were serum-starved for 48h (control) or cultured for the last 24h in the presence of MEK/PI3K inhibitors (UO/LY). MTT assay was performed to analyze cell viability. Values (n = 3) are shown as means ± SEM expressed as percentage of cell viability vs. control cells. No statistical difference was found
